# Supplementary material for: A low-cost fluorescence reader for in vitro transcription and nucleic acid detection with Cas13a
Source: PLoS One. 2019 Dec 18;14(12):e0220091. doi: 10.1371/journal.pone.0220091 (PMC6919979; doi:10.1371/journal.pone.0220091)
Supplement: S1 Appendix — (PDF) [file pone.0220091.s001.pdf]

S1 Appendix. Derivation of calibration function and analysis of measurement uncertainties.

Calibration function

In this section, we motivate Eq. (3), which we used for the calibration of our detector. We assume that the light intensity I at the LDR during a fluorescence measurement is a sum of signal intensity I_s and background intensity I_b:

$$I = I_s + I_b \tag{S1}$$

I_b corresponds to the intensity of the blank sample. With Eq. (1) this gives $R_b \propto I_b^{-\gamma}$ for the resistance of a blank sample and $R \propto (I_s + I_b)^{-\gamma}$ for the resistance of a fluorescent sample. Then, the normalized resistance can be expressed as

$$\frac{R(c)}{R_b} = \left(1 + \frac{I_s}{I_b}\right)^{-\gamma}. \tag{S2}$$

We assume that $I_s \propto I_0 c$ and $I_b \propto I_0$, where I_0 is the intensity of the excitation LED. Therefore,

$$\frac{I_s}{I_b} = k \cdot c, \tag{S3}$$

where k is a constant that depends on the spectral properties of the LED, the filter foils, the fluorophore, and the LDR. Importantly, k does not depend on I_0, i.e. the excitation light intensity. Inserting Eq. (S3) into Eq. (S2) relates R to c as

$$\frac{R(c)}{R_b} = (1 + kc)^{-\gamma}, \tag{S4}$$

where k and γ are the previously stated calibration parameters. After calibration, the inverse form of Eq. (S4) together with Eq. (2) is used to compute equivalent fluorescein concentrations c from resistances $R(U_{LDR})$

$$c = \frac{\left(\frac{R_b}{R(U_{LDR})}\right)^{\frac{1}{\gamma}} - 1}{k}, \tag{S5}$$

and resistance from measured voltages U_{LDR}

$$R = \frac{R_{ref}}{\frac{U_0}{U_{LDR}} - 1}. \tag{S6}$$

Measurement uncertainties

The total measurement uncertainty δc^{total} of a concentration measurement is given by the sum of the statistical and the systematic measurement uncertainty

$$\delta c = \delta c^{stat} + \delta c^{sys} \ , \tag{S7}$$

which are computed and saved by the detector software. In the following, we describe the derivation of expressions for δc^{stat} and δc^{sys}, which allows to make a general statement on confident measurement range of our device.

Statistical measurement uncertainties

First, we consider statistical uncertainties, which arise from fluctuations in the voltage measurements, probably caused by electromagnetic interference. The statistical uncertainty δU_{LDR}^{stat}, computed as the standard error of the mean of several measurements is propagated by

$$\delta c^{stat} = \left|\frac{\partial c}{\partial R}\right| \cdot \delta R^{stat} = \left|\frac{\partial c}{\partial R}\right| \left|\frac{\partial R}{\partial U_{LDR}}\right| \cdot \delta U_{LDR}^{stat} \ . \tag{S8}$$

With Eq. (S5) and (S6) we get

$$\left|\frac{\partial c}{\partial R}\right| = \left(\frac{R_b}{R}\right)^{\frac{1}{\gamma}} \cdot \frac{1}{\gamma} \cdot \frac{1}{k} \cdot \frac{1}{R} \tag{S9}$$

$$\left|\frac{\partial R}{\partial U_{LDR}}\right| = \frac{R_{ref}}{\frac{U_0}{U_{LDR}} - 1} \cdot \frac{\frac{U_0}{U_{LDR}}}{\frac{U_0}{U_{LDR}} - 1} \cdot \frac{U_0}{U_{LDR}} \cdot \frac{1}{U_0}, \tag{S10}$$

which gives an expression for the relative statistical uncertainty of the resistance R

$$\frac{\delta R^{stat}}{R} = \frac{U_0}{U_0 - U_{LDR}} \cdot \frac{\delta U_{LDR}^{stat}}{U_{LDR}} \tag{S11}$$

Then, the statistic uncertainty δc^{stat} of the concentration is given by

$$\delta c^{stat} = \left(\frac{R_b}{R}\right)^{\frac{1}{\gamma}} \frac{1}{\gamma}\frac{1}{k} \cdot \frac{\delta R^{stat}}{R} \ . \tag{S12}$$

As δU_{LDR}^{stat} is computed as the standard error of the mean, the statistical uncertainty can be reduced by repeating the voltage measurements many times. In our setting, taking 50 voltage measurements per data point was sufficient to reduce the relative statistical uncertainty $\frac{\delta c^{stat}}{c}$ to $< 0.2\%$ for all concentrations measured. As this is small compared to the systematic uncertainty estimated in the following section, we will omit δc^{stat} in the following and identify δc with δc^{sys}.

Systematic measurement uncertainties

To compute the relative systematic uncertainty $\frac{\delta c}{c}$ as a function of the concentration c, we consider the following uncertainties:

- δR_b, which is the standard error of the mean of R_b.
- $\delta \lambda$ and δk, which are the asymptotic standard errors obtained from fitting λ and k

- δU_{LDR} and δU_0, which correspond to a 1 digit accuracy of measuring U_{LDR} and U_0 with the microcontroller

The uncertainties $\delta\gamma$, δk and δR_b are generally dependent on each other as they where obtained from the same fit. We therefore compute the total uncertainty δc, in accordance to [1][p.79], by absolute addition of the individual uncertainties:

$$\delta c = \left|\frac{\partial c}{\partial \gamma}\right| \cdot \delta\gamma + \left|\frac{\partial c}{\partial R_b}\right| \cdot \delta R_b + \left|\frac{\partial c}{\partial R}\frac{\partial R}{\partial U_{LDR}}\right| \cdot \delta U_{LDR} + \left|\frac{\partial c}{\partial R}\frac{\partial R}{\partial U_0}\right| \cdot \delta U_0 + \left|\frac{\partial c}{\partial k}\cdot\right| \delta k \quad \text{(S13)}$$

Computation of the absolute values of the partial derivatives from Eq. (S5) and (S6) gives

$$\left|\frac{\partial c}{\partial \gamma}\right| = \left(\frac{R_b}{R}\right)^{\frac{1}{\gamma}} \cdot \ln\left(\frac{R_b}{R}\right)^{\frac{1}{\gamma}} \cdot \frac{1}{\gamma} \cdot \frac{1}{k} \quad \text{(S14)}$$

$$\left|\frac{\partial c}{\partial R_b}\right| = \left(\frac{R_b}{R}\right)^{\frac{1}{\gamma}} \cdot \frac{1}{\gamma} \cdot \frac{1}{k} \cdot \frac{1}{R_b} \quad \text{(S15)}$$

$$\left|\frac{\partial c}{\partial R}\right| = \left(\frac{R_b}{R}\right)^{\frac{1}{\gamma}} \cdot \frac{1}{\gamma} \cdot \frac{1}{k} \cdot \frac{1}{R} \quad \text{(S16)}$$

$$\left|\frac{\partial R}{\partial U_{LDR}}\right| = \frac{R_{ref}}{\frac{U_0}{U_{LDR}} - 1} \cdot \frac{\frac{U_0}{U_{LDR}}}{\frac{U_0}{U_{LDR}} - 1} \cdot \frac{U_0}{U_{LDR}} \cdot \frac{1}{U_0} \quad \text{(S17)}$$

$$\left|\frac{\partial R}{\partial U_0}\right| = \frac{R_{ref}}{\frac{U_0}{U_{LDR}} - 1} \cdot \frac{\frac{U_0}{U_{LDR}}}{\frac{U_0}{U_{LDR}} - 1} \cdot \frac{1}{U_0} \quad \text{(S18)}$$

$$\left|\frac{\partial c}{\partial k}\right| = \frac{\left(\frac{R_b}{R}\right)^{\frac{1}{\gamma}} - 1}{k} \cdot \frac{1}{k}\ , \quad \text{(S19)}$$

where we assume $R < R_b$ to account for the absolute values.

We identify two reoccurring expressions in the partial derivatives, which can be rewritten in terms of the concentration c by using rearranged forms of Eq. (S5) and (S6):

$$\left(\frac{R_b}{R}\right)^{\frac{1}{\gamma}} = 1 + kc \quad \text{(S20)}$$

$$\frac{U_0}{U_{LDR}} = 1 + \frac{R_{ref}}{R} \quad \text{(S21)}$$

With this the partial derivatives can be expressed as

$$\left|\frac{\partial c}{\partial \gamma}\right| = (1+kc)\cdot \ln(1+kc)\cdot \frac{1}{\gamma}\cdot\frac{1}{k} \tag{S22}$$

$$\left|\frac{\partial c}{\partial R_b}\right| = (1+kc)\cdot \frac{1}{\gamma}\cdot\frac{1}{k}\cdot\frac{1}{R_b} \tag{S23}$$

$$\left|\frac{\partial c}{\partial R}\right| = (1+kc)\cdot \frac{1}{\gamma}\cdot\frac{1}{k}\cdot\frac{1}{R} \tag{S24}$$

$$\left|\frac{\partial R}{\partial U_{LDR}}\right| = R\cdot\frac{(R+R_{ref})^2}{R_{ref}R}\cdot\frac{1}{U_0} \tag{S25}$$

$$\left|\frac{\partial R}{\partial U_0}\right| = R\cdot\frac{R+R_{ref}}{R_{ref}}\cdot\frac{1}{U_0} \tag{S26}$$

$$\left|\frac{\partial c}{\partial k}\right| = c\cdot\frac{1}{k}\ , \tag{S27}$$

which inserted in Eq. (S13) gives

$$\delta c = \left(\frac{1+kc}{k}\right)\left(\ln(1+kc)\frac{\delta\gamma}{\gamma} + \frac{1}{\gamma}\frac{\delta R_b}{R_b} + \frac{1}{\gamma}\left(\frac{R}{R_{ref}}+1\right)\left(2+\frac{R_{ref}}{R}\right)\frac{1}{U_0}\right) + c\frac{\delta k}{k}\ . \tag{S28}$$

Inserting Eq. (S6) for R and dividing by c gives the final equation for the relative systematic uncertainty:

$$\begin{aligned}\frac{\delta c}{c} = &\left(\frac{1}{kc}+1\right)\left(\ln(1+kc)\frac{\delta\gamma}{\gamma} + \frac{1}{\gamma}\frac{\delta R_b}{R_b}\right.\\ &\left.+\frac{1}{\gamma}\left(\frac{R_b}{R_{ref}}(1+kc)^{-\gamma}+1\right)\left(2+\frac{R_{ref}}{R_b}(1+kc)^{\gamma}\right)\frac{1}{U_0}\right) + \frac{\delta k}{k}\ .\end{aligned} \tag{S29}$$

For determination of the confident measuring range we require $\frac{\delta c}{c} < 0.15$ for the relative systematic uncertainty. We obtained [9 nM : 1730 nM] for the confident measurement range by numerical root finding.

References

1. Taylor J. Introduction to error analysis, the study of uncertainties in physical measurements. University Science Books; 1997.
