# Supplementary figures and images for: A low-cost fluorescence reader for in vitro transcription and nucleic acid detection with Cas13a
Source: PLoS One. 2019 Dec 18;14(12):e0220091. doi: 10.1371/journal.pone.0220091 (PMC6919979; doi:10.1371/journal.pone.0220091)

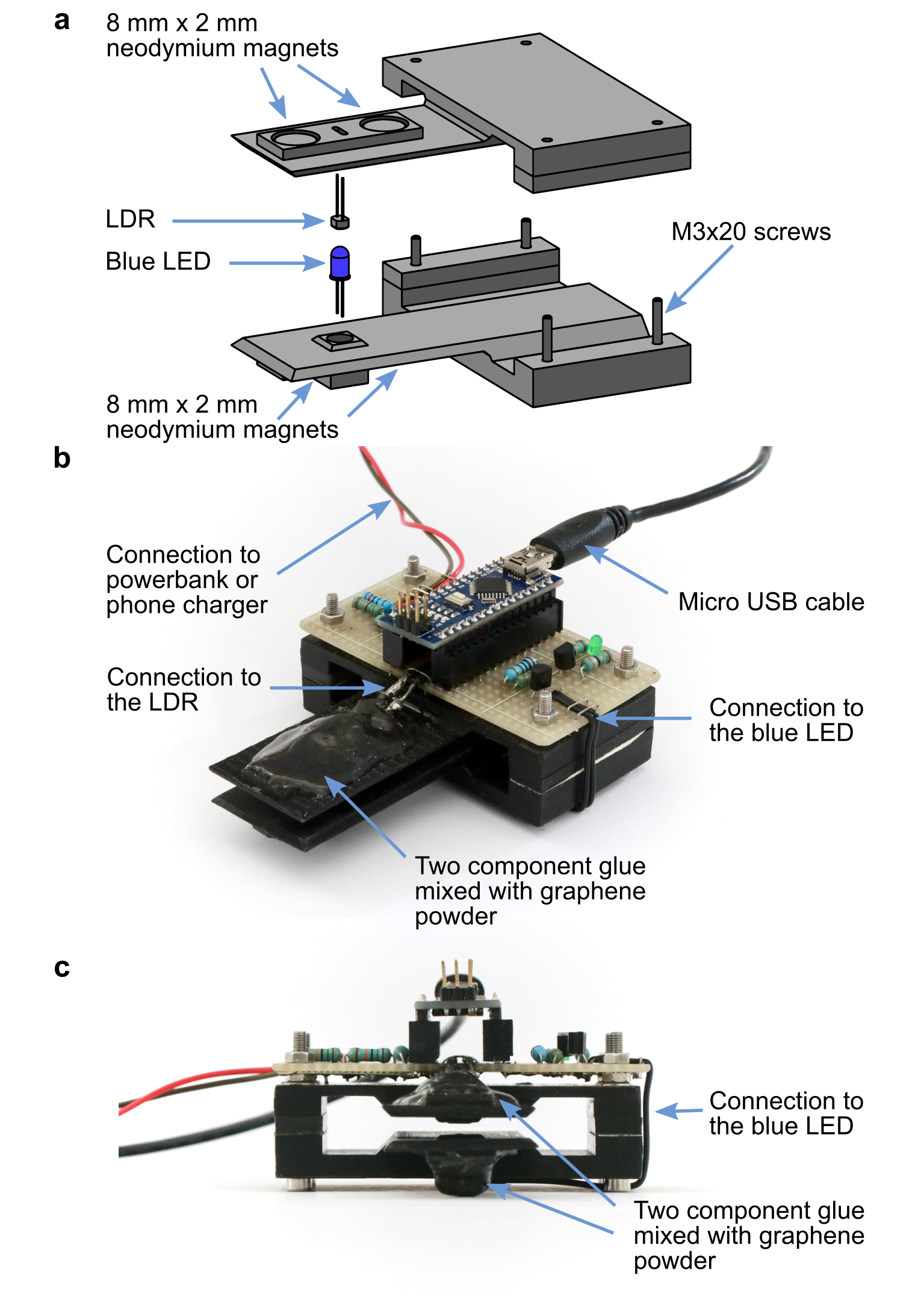

Supplement: S1 Fig — (a) CAD drawing, (b) side view photograph and (c) front view photograph illustrating the assembly of the detection unit. The 3D printed parts are first treated with sand paper and the holes for the screws are drilled. Magnets, LED and LDR are inserted and glued into the corresponding cavities. For the LED and LDR we mixed the glue with graphene to block transmission of background light. Then the circuit board carrying the microcontroller (S3 Fig) is assembled on top of the unit and fixed with screws. Finally, the LED and LDR are connected to the circuit board. (PNG) [file pone.0220091.s002.png]

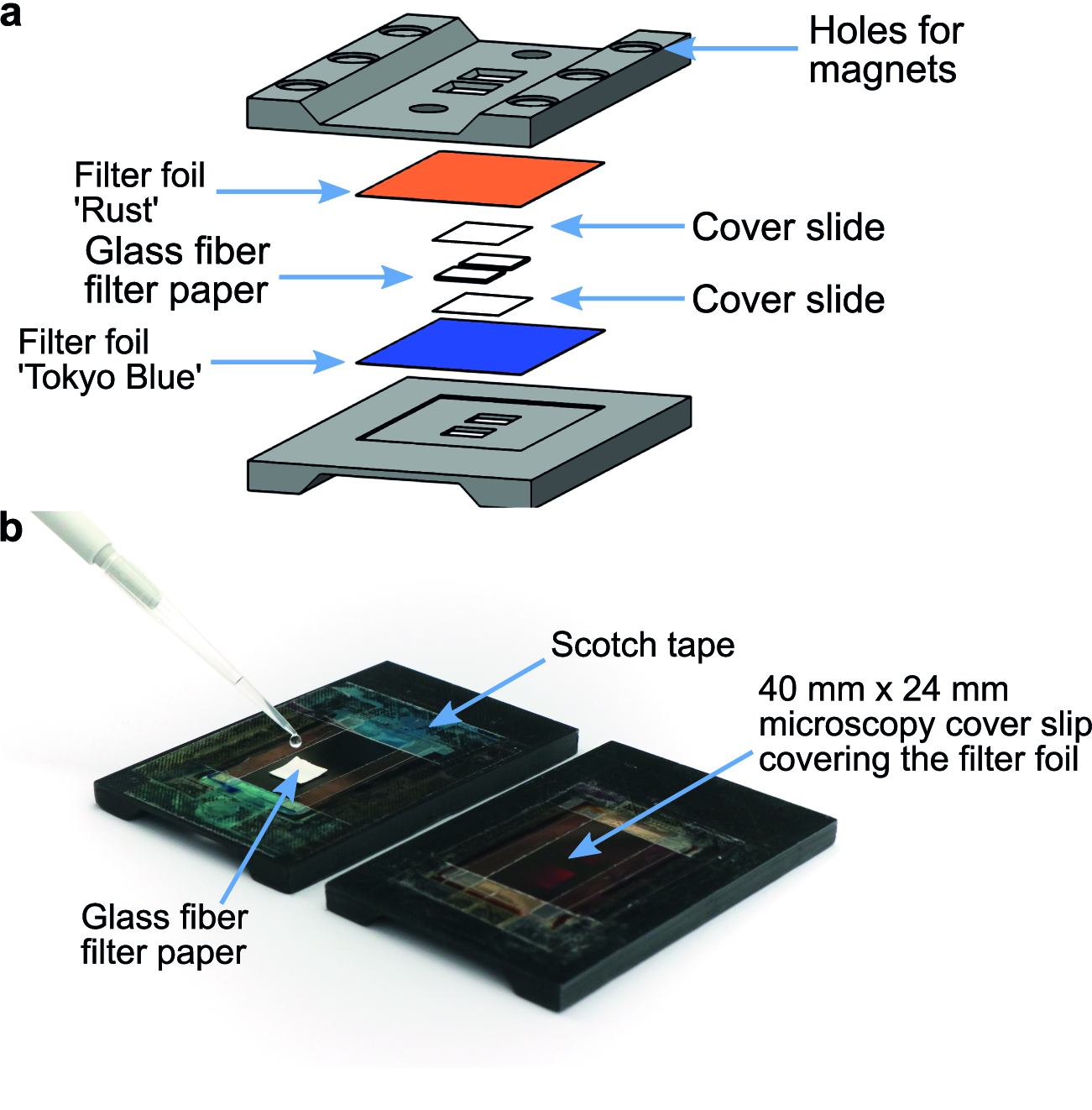

Supplement: S2 Fig — (a) CAD drawing and (b) photograph illustrating the assembly of the assay cartridge. The 3D printed parts are first treated with sand paper. Then the magnets are glued into the corresponding cavities. The filter foils and protective cover slides are cut into the appropriate size, assembled in front of the detection window and fixed with Scotch tape. The filter foils are covered with microscope cover slides to facilitate cleaning with ethanol and water between measurements. To obtain a clean optical pathway, the transmission windows must be free of Scotch tape. A piece of filter paper carrying the sample is placed in front of the transmission window. (TIF) [file pone.0220091.s003.tif]

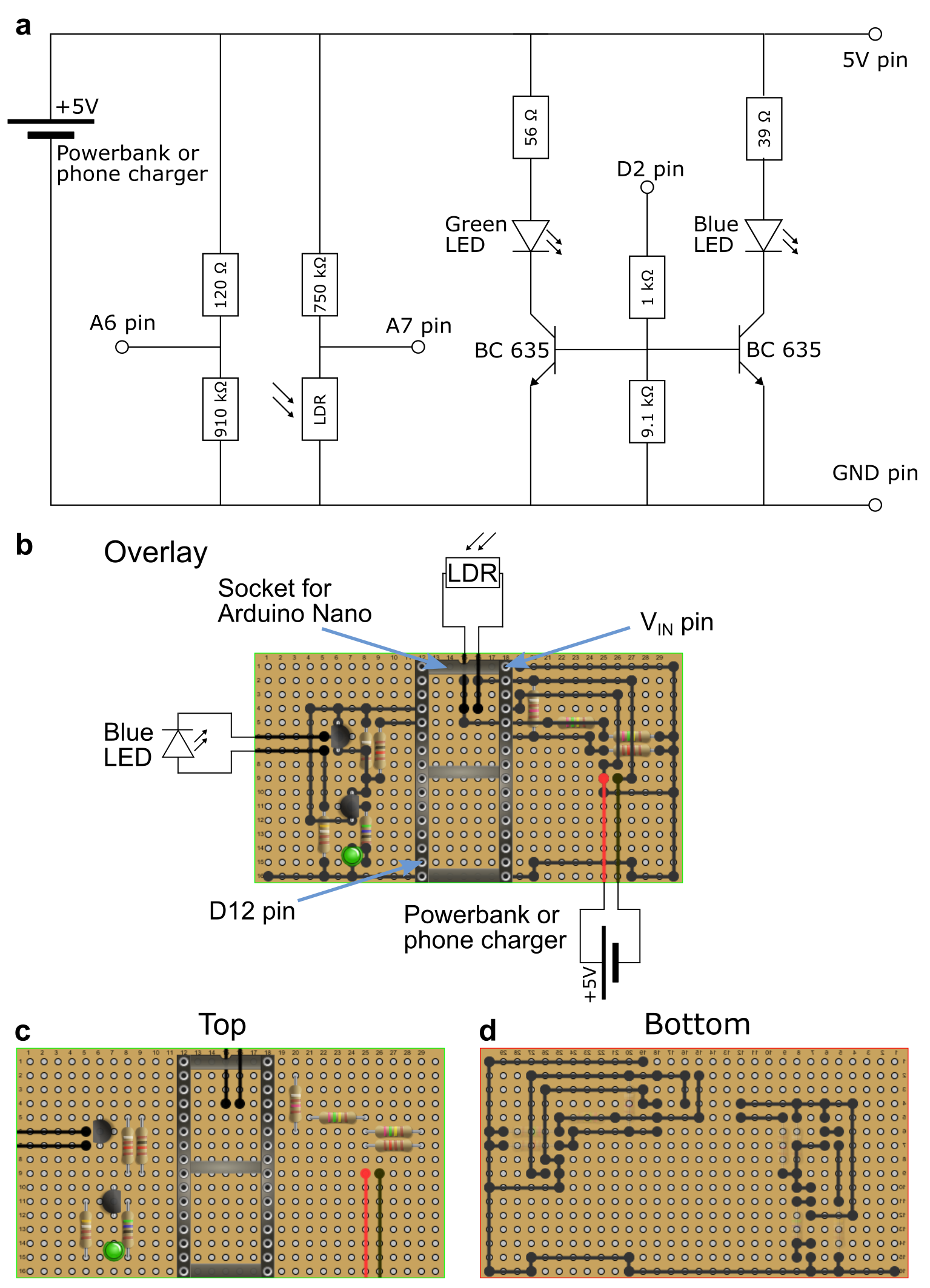

Supplement: S3 Fig — (a) The blue excitation light LED and a green status LED are controlled by NPN transistors via the digital pin-out of the microcontroller. The resistance of the LDR changes according to the intensity of incoming emission light and is measured via a voltage divider using an analog input-pin of the microcontroller. (b) overlay, (c) top, and (d) bottom view of the used circuit board layout for soldering. (PNG) [file pone.0220091.s004.png]

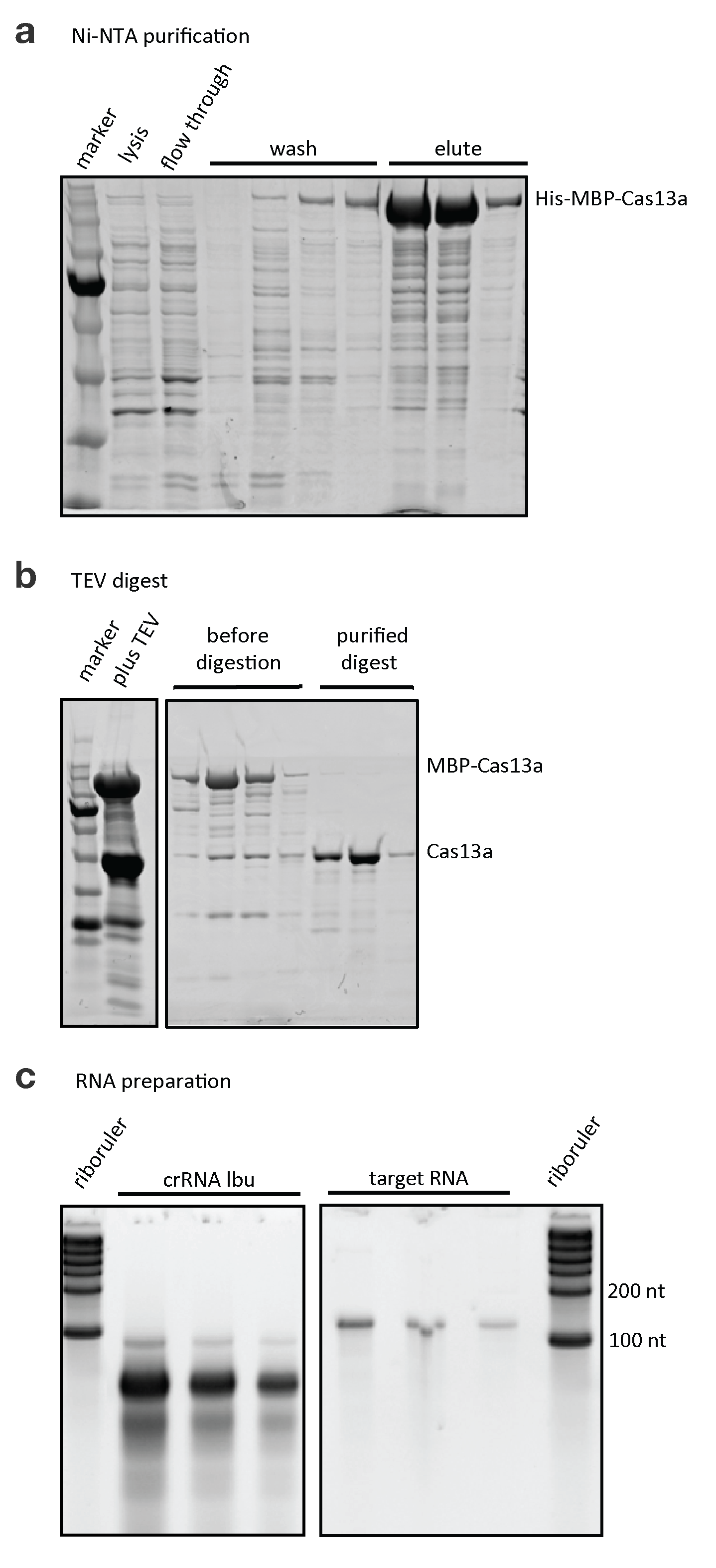

Supplement: S4 Fig — SDS-gels of Cas13a Ni-NTA purification after cell lysis (a) and after TEV protease digestion (b). (c) Gel-electrophoretic analysis of In vitro crRNA and targetRNA transcription for Cas13a assay. (PNG) [file pone.0220091.s005.png]

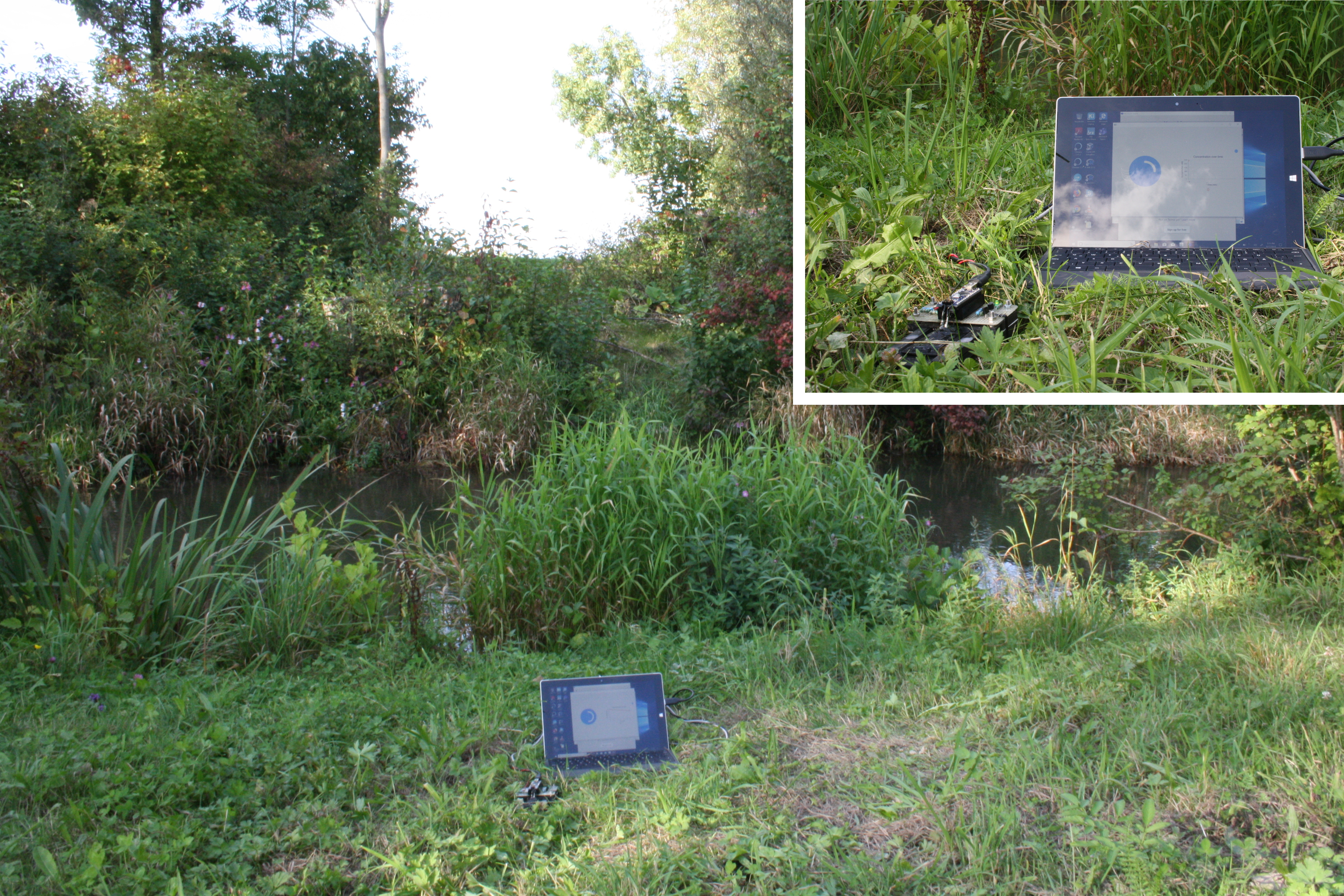

Supplement: S5 Fig — The detector can be operated and powered from a Windows tablet. (JPG) [file pone.0220091.s006.jpg]

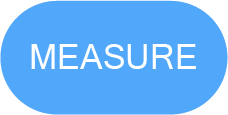

Supplement: S1 File — Are available via Github: https://github.com/Katzi93/Fluorescence_detector. (ZIP) [file pone.0220091.s007.zip › S1 File/software.Lightbringer/src/resources/Measure_on.png]

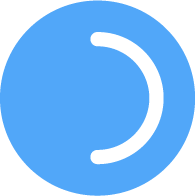

Supplement: S1 File — Are available via Github: https://github.com/Katzi93/Fluorescence_detector. (ZIP) [file pone.0220091.s007.zip › S1 File/software.Lightbringer/src/resources/Loading_05.png]

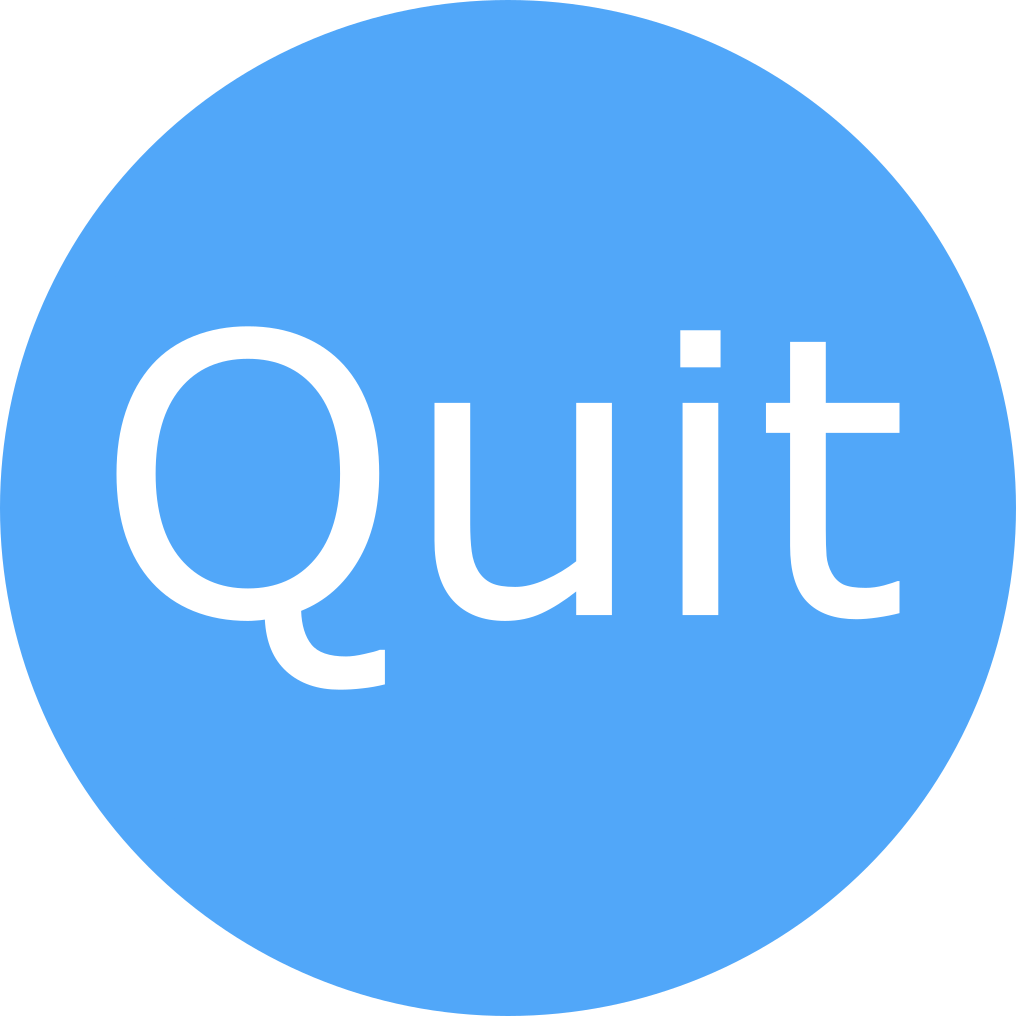

Supplement: S1 File — Are available via Github: https://github.com/Katzi93/Fluorescence_detector. (ZIP) [file pone.0220091.s007.zip › S1 File/software.Lightbringer/src/resources/Quit.png]

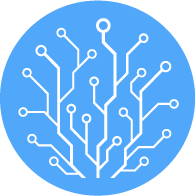

Supplement: S1 File — Are available via Github: https://github.com/Katzi93/Fluorescence_detector. (ZIP) [file pone.0220091.s007.zip › S1 File/software.Lightbringer/src/resources/ON_OFF_1.png]

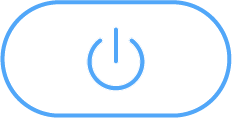

Supplement: S1 File — Are available via Github: https://github.com/Katzi93/Fluorescence_detector. (ZIP) [file pone.0220091.s007.zip › S1 File/software.Lightbringer/src/resources/Power_off_v2.png]

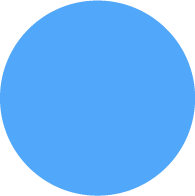

Supplement: S1 File — Are available via Github: https://github.com/Katzi93/Fluorescence_detector. (ZIP) [file pone.0220091.s007.zip › S1 File/software.Lightbringer/src/resources/Loading_00.png]

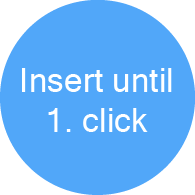

Supplement: S1 File — Are available via Github: https://github.com/Katzi93/Fluorescence_detector. (ZIP) [file pone.0220091.s007.zip › S1 File/software.Lightbringer/src/resources/Click_1.png]

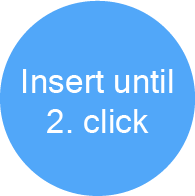

Supplement: S1 File — Are available via Github: https://github.com/Katzi93/Fluorescence_detector. (ZIP) [file pone.0220091.s007.zip › S1 File/software.Lightbringer/src/resources/Click_2.png]

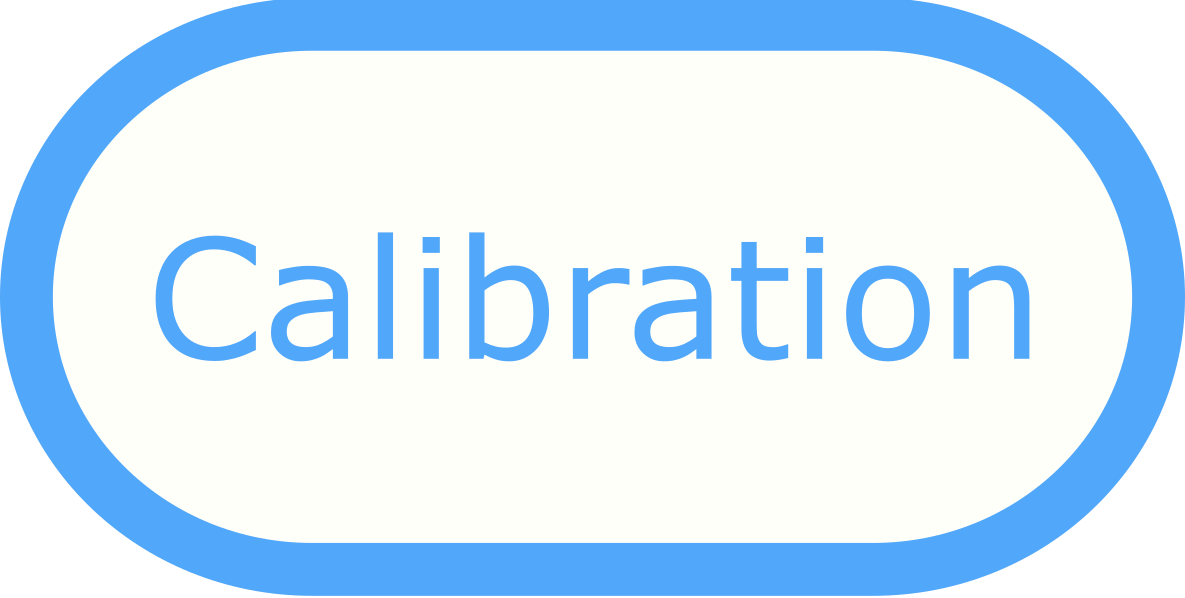

Supplement: S1 File — Are available via Github: https://github.com/Katzi93/Fluorescence_detector. (ZIP) [file pone.0220091.s007.zip › S1 File/software.Lightbringer/src/resources/Calibration_click.png]

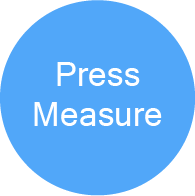

Supplement: S1 File — Are available via Github: https://github.com/Katzi93/Fluorescence_detector. (ZIP) [file pone.0220091.s007.zip › S1 File/software.Lightbringer/src/resources/Measure_1.png]

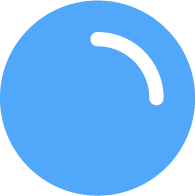

Supplement: S1 File — Are available via Github: https://github.com/Katzi93/Fluorescence_detector. (ZIP) [file pone.0220091.s007.zip › S1 File/software.Lightbringer/src/resources/Loading_025.png]

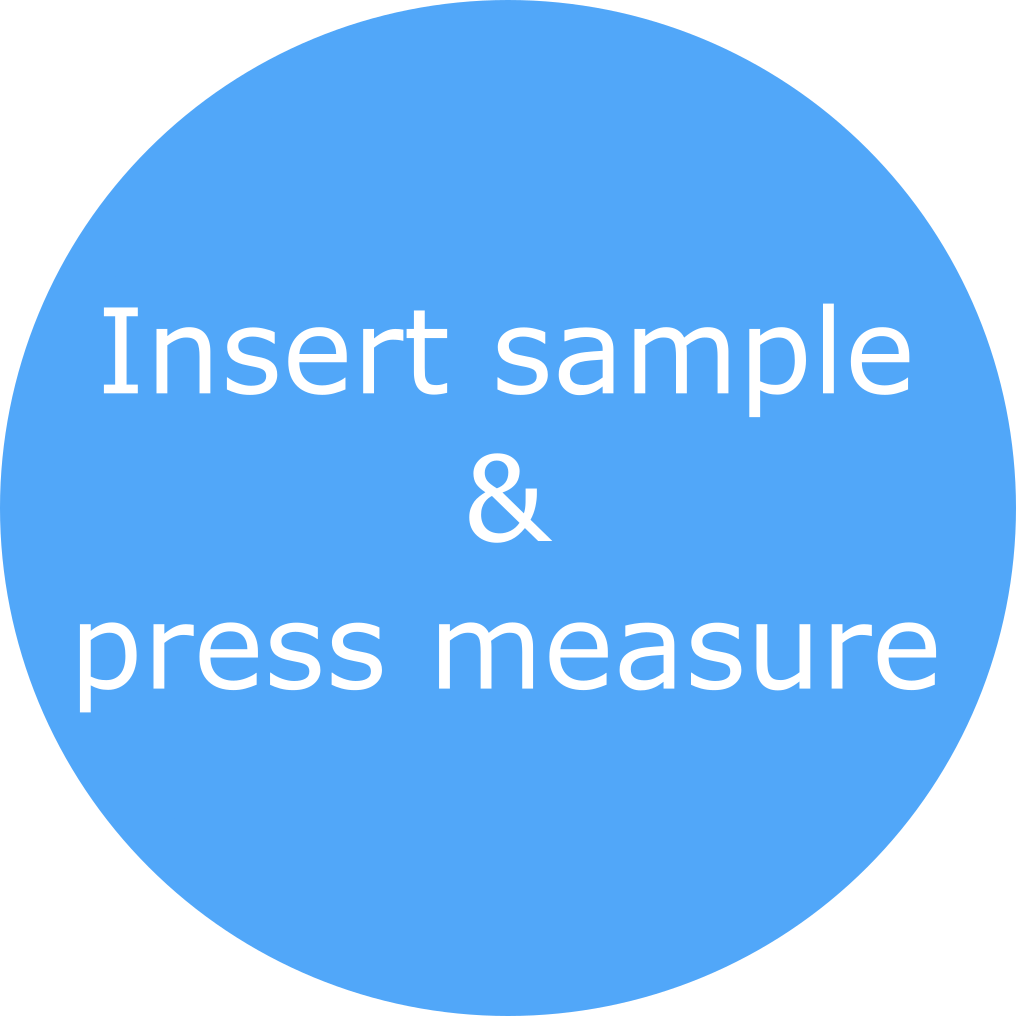

Supplement: S1 File — Are available via Github: https://github.com/Katzi93/Fluorescence_detector. (ZIP) [file pone.0220091.s007.zip › S1 File/software.Lightbringer/src/resources/status_sample.png]

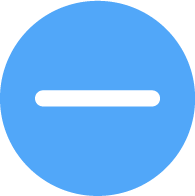

Supplement: S1 File — Are available via Github: https://github.com/Katzi93/Fluorescence_detector. (ZIP) [file pone.0220091.s007.zip › S1 File/software.Lightbringer/src/resources/Negative.png]

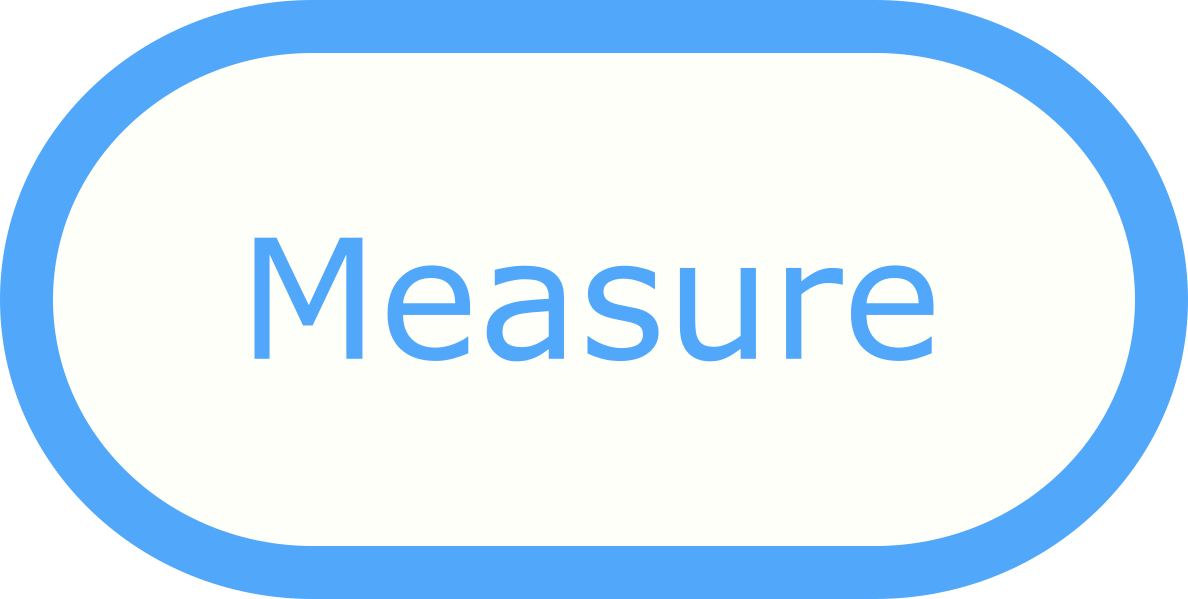

Supplement: S1 File — Are available via Github: https://github.com/Katzi93/Fluorescence_detector. (ZIP) [file pone.0220091.s007.zip › S1 File/software.Lightbringer/src/resources/Measure_click.png]

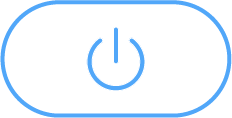

Supplement: S1 File — Are available via Github: https://github.com/Katzi93/Fluorescence_detector. (ZIP) [file pone.0220091.s007.zip › S1 File/software.Lightbringer/src/resources/Power_off.png]

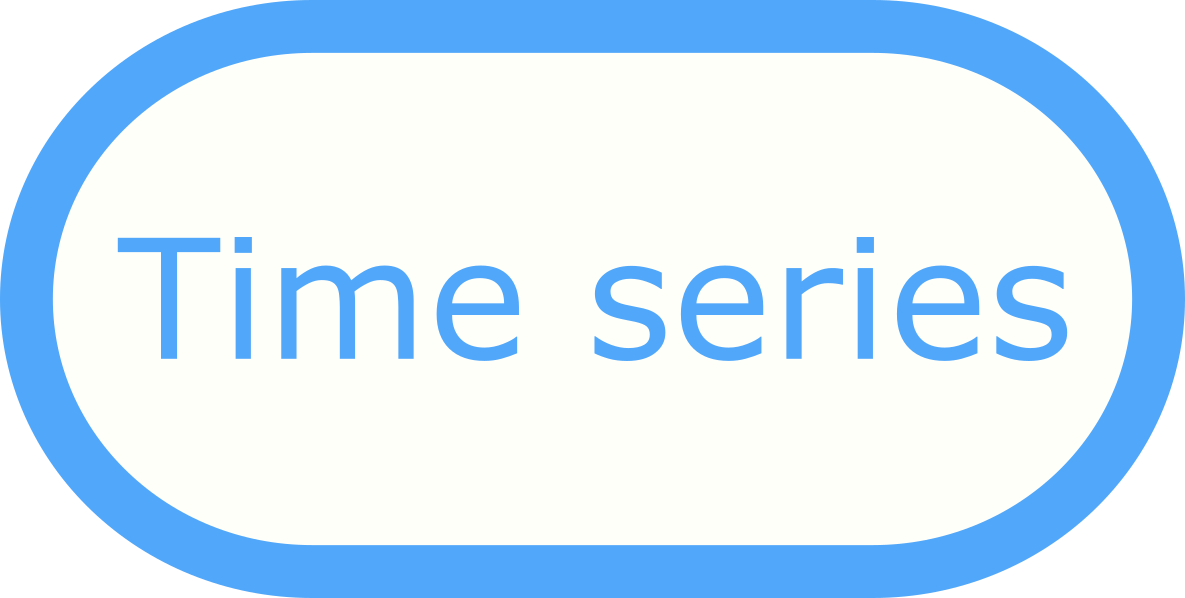

Supplement: S1 File — Are available via Github: https://github.com/Katzi93/Fluorescence_detector. (ZIP) [file pone.0220091.s007.zip › S1 File/software.Lightbringer/src/resources/Timeseries_click.png]

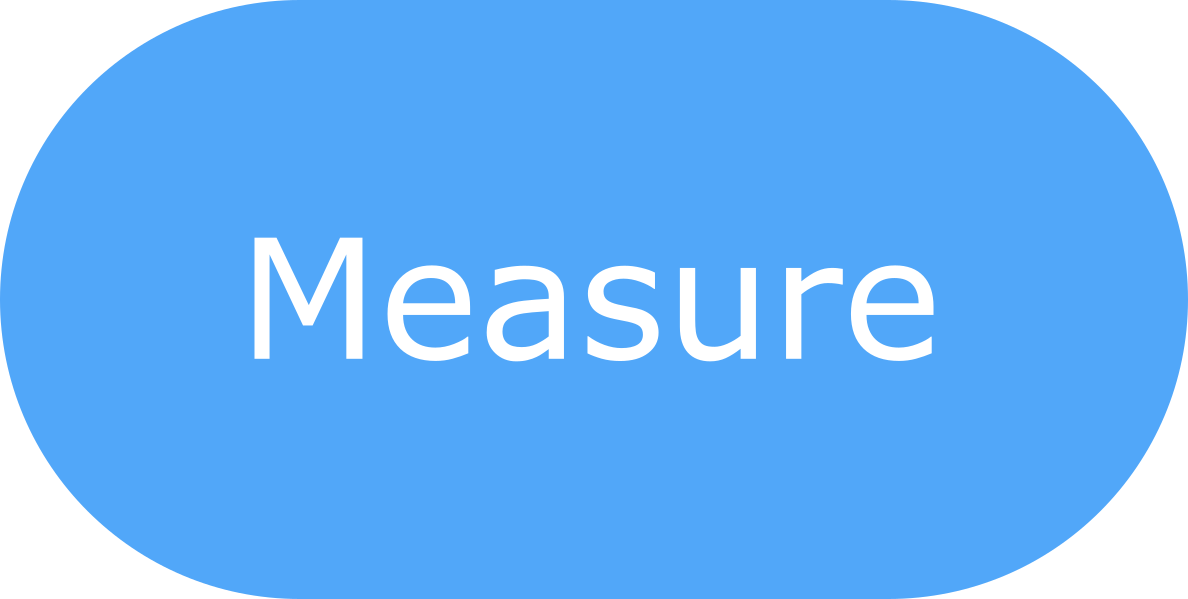

Supplement: S1 File — Are available via Github: https://github.com/Katzi93/Fluorescence_detector. (ZIP) [file pone.0220091.s007.zip › S1 File/software.Lightbringer/src/resources/Measure.png]

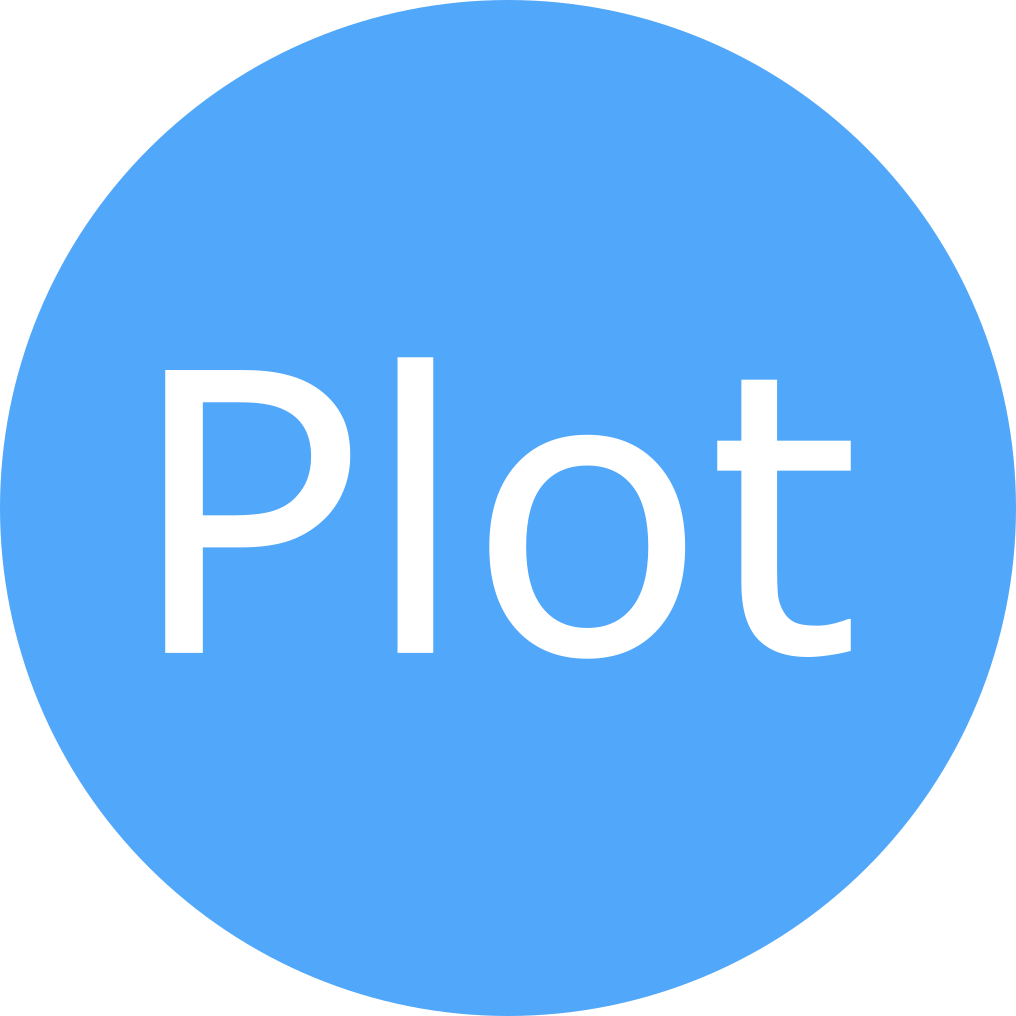

Supplement: S1 File — Are available via Github: https://github.com/Katzi93/Fluorescence_detector. (ZIP) [file pone.0220091.s007.zip › S1 File/software.Lightbringer/src/resources/plot.png]

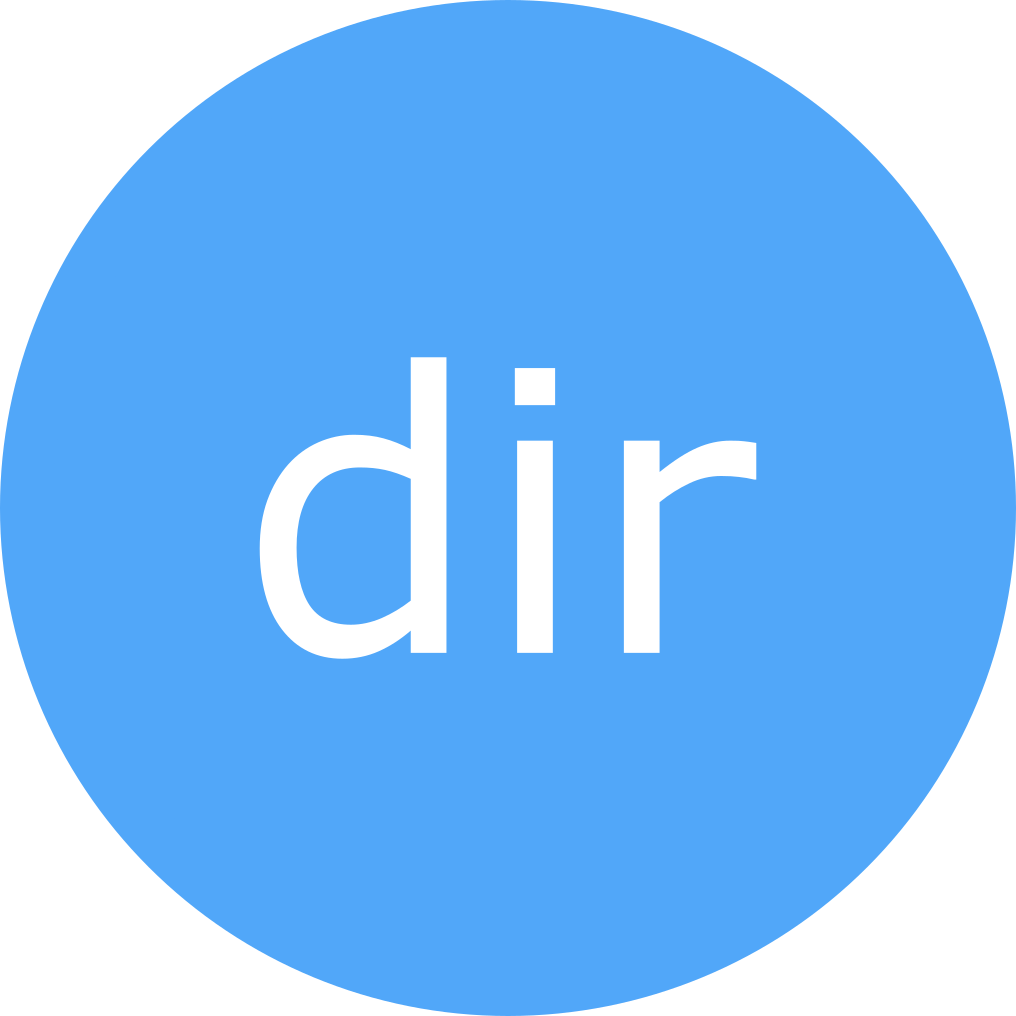

Supplement: S1 File — Are available via Github: https://github.com/Katzi93/Fluorescence_detector. (ZIP) [file pone.0220091.s007.zip › S1 File/software.Lightbringer/src/resources/cd.png]

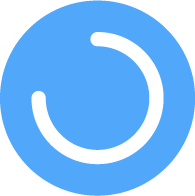

Supplement: S1 File — Are available via Github: https://github.com/Katzi93/Fluorescence_detector. (ZIP) [file pone.0220091.s007.zip › S1 File/software.Lightbringer/src/resources/Loading_075.png]

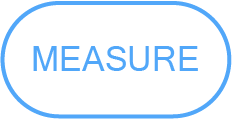

Supplement: S1 File — Are available via Github: https://github.com/Katzi93/Fluorescence_detector. (ZIP) [file pone.0220091.s007.zip › S1 File/software.Lightbringer/src/resources/Measure_off.png]

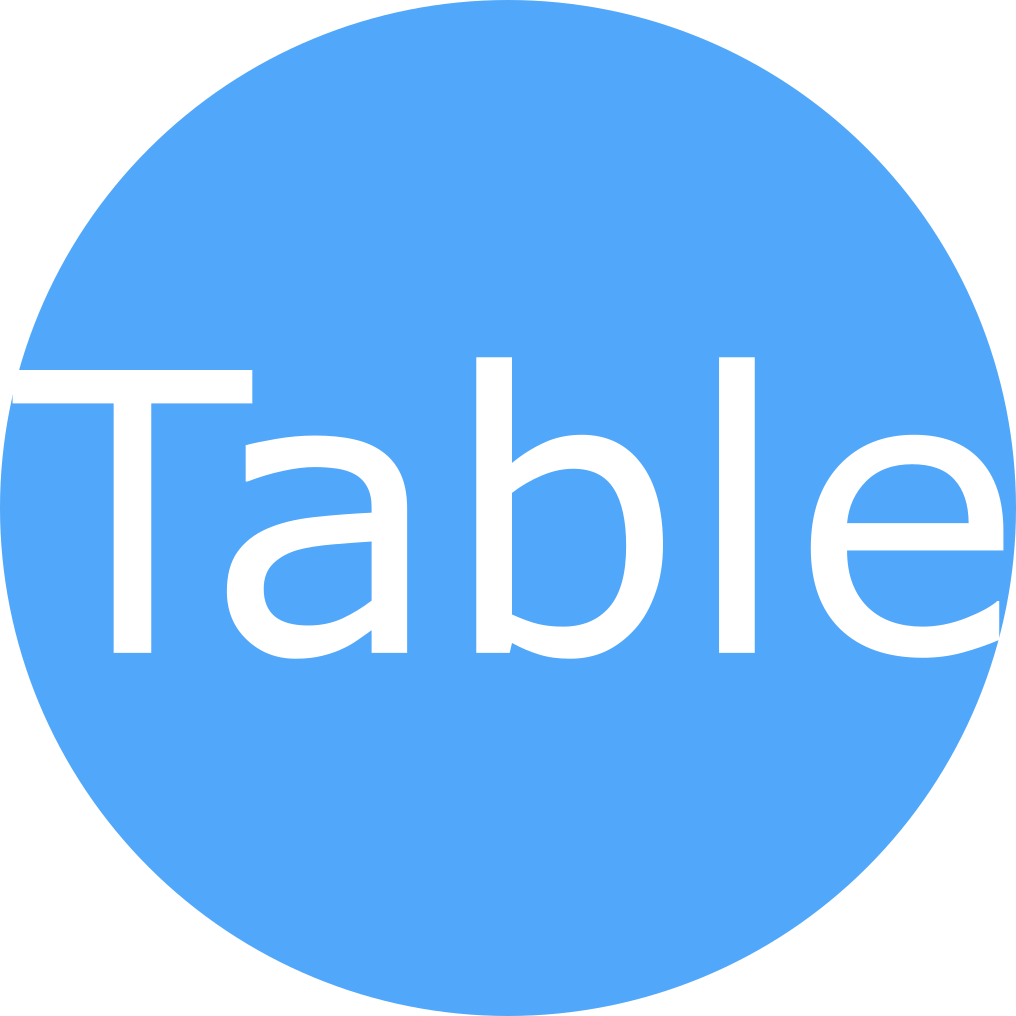

Supplement: S1 File — Are available via Github: https://github.com/Katzi93/Fluorescence_detector. (ZIP) [file pone.0220091.s007.zip › S1 File/software.Lightbringer/src/resources/table.png]

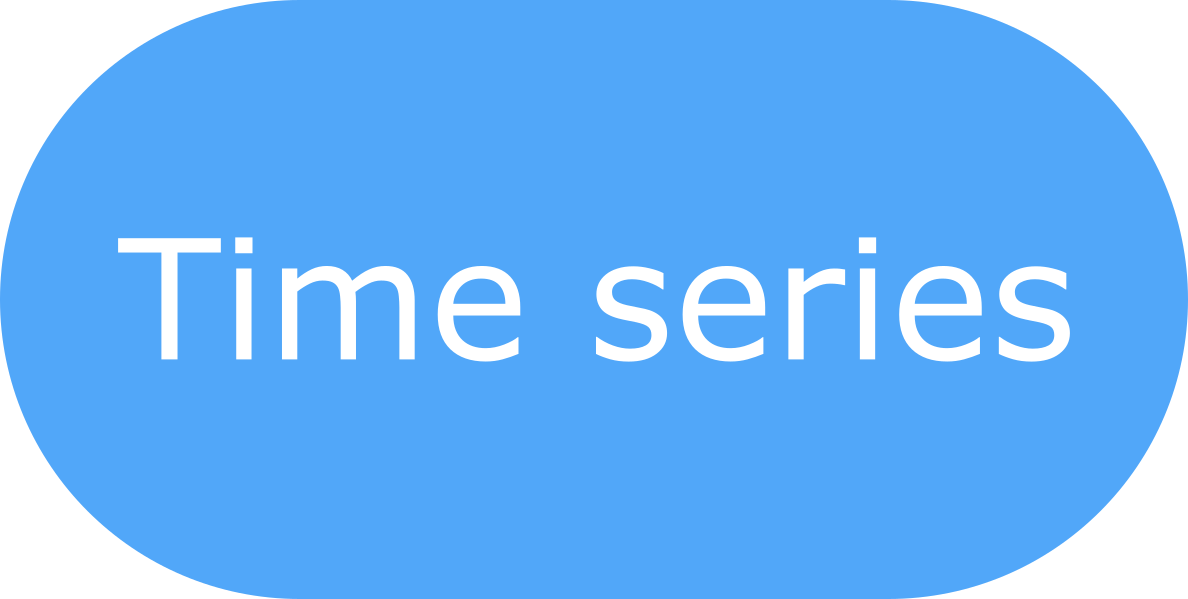

Supplement: S1 File — Are available via Github: https://github.com/Katzi93/Fluorescence_detector. (ZIP) [file pone.0220091.s007.zip › S1 File/software.Lightbringer/src/resources/Timeseries.png]

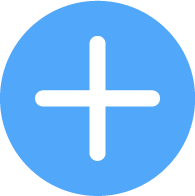

Supplement: S1 File — Are available via Github: https://github.com/Katzi93/Fluorescence_detector. (ZIP) [file pone.0220091.s007.zip › S1 File/software.Lightbringer/src/resources/Positive.png]

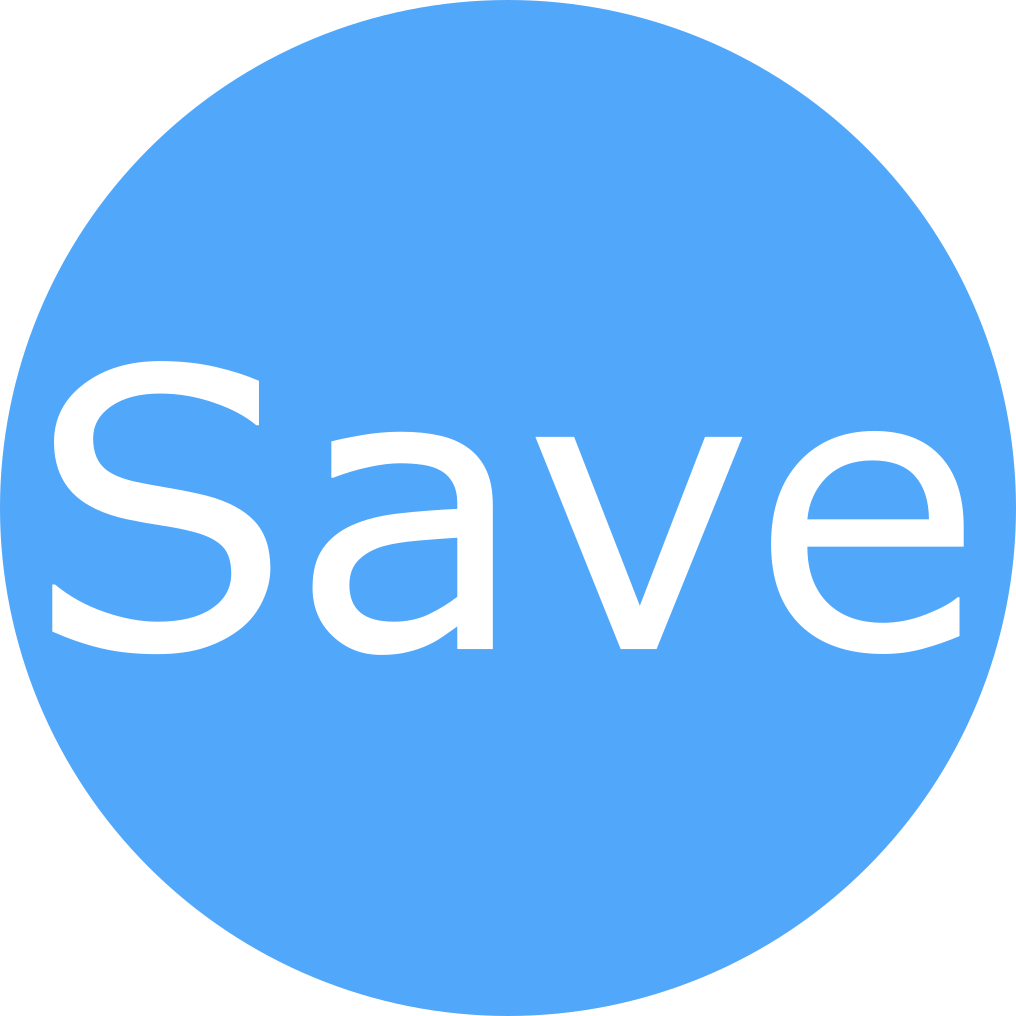

Supplement: S1 File — Are available via Github: https://github.com/Katzi93/Fluorescence_detector. (ZIP) [file pone.0220091.s007.zip › S1 File/software.Lightbringer/src/resources/save.png]

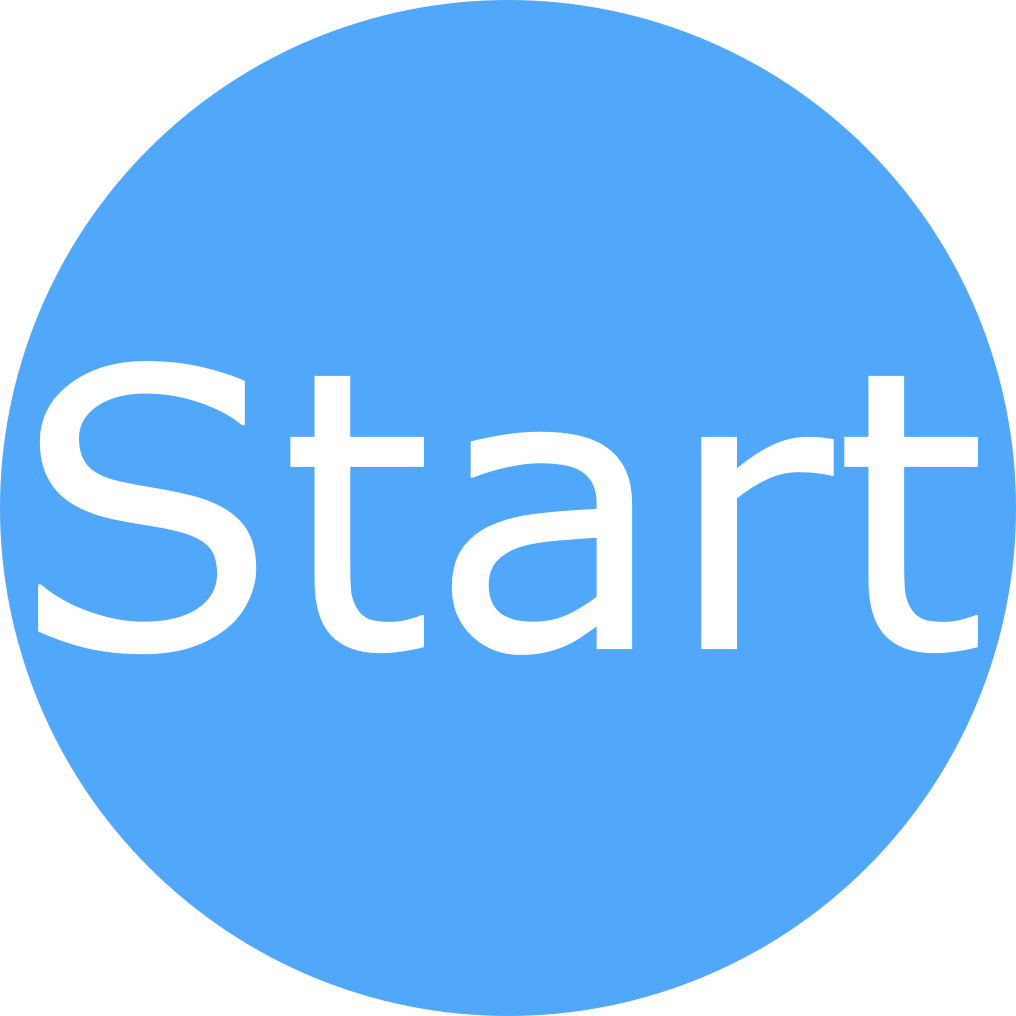

Supplement: S1 File — Are available via Github: https://github.com/Katzi93/Fluorescence_detector. (ZIP) [file pone.0220091.s007.zip › S1 File/software.Lightbringer/src/resources/start.png]

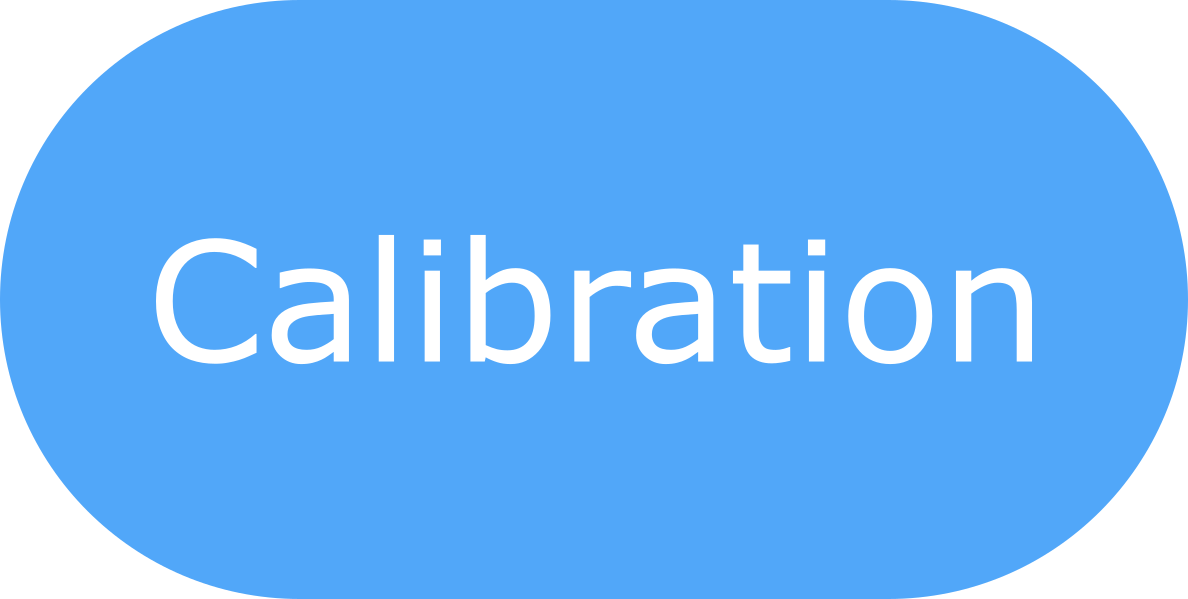

Supplement: S1 File — Are available via Github: https://github.com/Katzi93/Fluorescence_detector. (ZIP) [file pone.0220091.s007.zip › S1 File/software.Lightbringer/src/resources/Calibration.png]

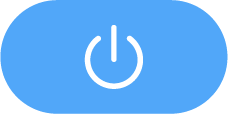

Supplement: S1 File — Are available via Github: https://github.com/Katzi93/Fluorescence_detector. (ZIP) [file pone.0220091.s007.zip › S1 File/software.Lightbringer/src/resources/Power_on.png]

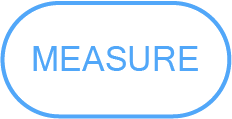

Supplement: S1 File — Are available via Github: https://github.com/Katzi93/Fluorescence_detector. (ZIP) [file pone.0220091.s007.zip › S1 File/software.Lightbringer/src/resources/Measure_off_v2.png]
