# Supplementary material for: A low-cost fluorescence reader for in vitro transcription and nucleic acid detection with Cas13a
Source: PLoS One. 2019 Dec 18;14(12):e0220091. doi: 10.1371/journal.pone.0220091 (PMC6919979; doi:10.1371/journal.pone.0220091)
Supplement: S3 File — Lbu-Cas13a, iSpinach, crRNA and Cas13a. (PDF) [file pone.0220091.s009.pdf]

p2CT-His-MBU-Lbu_C13a_WT
https://benchling.com/s/seq-iiOl2ZpMc8ZHUZwu1GRj
T7 promoter, 6x His, TEV cleavage site, Lbu Cas13a, T7 terminator

---

TAATACGACTCACTATAGGGAGACCACAACGGTTTCCCTCTAGTGCCGGCT
CCGGAGAGCTCTTTAATTAAGCGGCCGCCCTGCAGGACTCGAGTTCTAGA
AATAATTTTGTTTAACTTTAAGAAGGAGATATACATATGAAATCTTCTCACCA
TCACCATCACCATGGTTCTTCTATGAAAATCGAAGAAGGTAAACTGGTAATC
TGGATTAACGGCGATAAAGGCTATAACGGTCTCGCTGAAGTCGGTAAGAAA
TTCGAGAAAGATACCGGAATTAAAGTCACCGTTGAGCATCCGGATAAACTG
GAAGAGAAATTCCCACAGGTTGCGGCAACTGGCGATGGCCCTGACATTAT
CTTCTGGGCACACGACCGCTTTGGTGGCTACGCTCAATCTGGCCTGTTGG
CTGAAATCACCCCGGACAAAGCGTTCCAGGACAAGCTGTATCCGTTTACCT
GGGATGCCGTACGTTACAACGGCAAGCTGATTGCTTACCCGATCGCTGTT
GAAGCGTTATCGCTGATTTATAACAAAGATCTGCTGCCGAACCCGCCAAAA
ACCTGGGAAGAGATCCCGGCGCTGGATAAAGAACTGAAAGCGAAAGGTAA
GAGCGCGCTGATGTTCAACCTGCAAGAACCGTACTTCACCTGGCCGCTGA
TTGCTGCTGACGGGGGTTATGCGTTCAAGTATGAAAACGGCAAGTACGAC
ATTAAAGACGTGGGCGTGGATAACGCTGGCGCGAAAGCGGGTCTGACCTT
CCTGGTTGACCTGATTAAAAACAAACACATGAATGCAGACACCGATTACTC
CATCGCAGAAGCTGCCTTTAATAAAGGCGAAACAGCGATGACCATCAACG
GCCCGTGGGCATGGTCCAACATCGACACCAGCAAAGTGAATTATGGTGTA
ACGGTACTGCCGACCTTCAAGGGTCAACCATCCAAACCGTTCGTTGGCGT
GCTGAGCGCAGGTATTAACGCCGCCAGTCCGAACAAAGAGCTGGCAAAA
GAGTTCCTCGAAAACTATCTGCTGACTGATGAAGGTCTGGAAGCGGTTAAT
AAAGACAAACCGCTGGGTGCCGTAGCGCTGAAGTCTTACGAGGAAGAGTT
GGCGAAAGATCCACGTATTGCCGCCACTATGGAAAACGCCCAGAAAGGTG
AAATCATGCCGAACATCCCGCAGATGTCCGCTTTCTGGTATGCCGTGCGTA
CTGCGGTGATCAACGCCGCCAGCGGTCGTCAGACTGTCGATGAAGCCCT
GAAAGACGCGCAGACTAATTCGAGCTCGAACAACAACAACAATAACAATAA
CAACAACCTCGGGATCGAGGAAAACCTGTACTTCCAATCCAATgcaATGAA
AGTGACGAAGGTAGGAGGCATTTCGCATAAGAAGTACACGTCCGAAGGCC
GCTTAGTGAAGTCAGAATCGGAAGAAAATCGCACAGACGAACGTCTGTCG
GCGTTGCTTAATATGCGCCTTGACATGTATATCAAGAATCCCAGCAGCACG
GAAACCAAGGAAAATCAAAAACGCATTGGGAAATTAAAGAAATTCTTCTCAA
ACAAAATGGTCTATCTTAAAGACAATACCTTGAGTTTGAAGAATGGAAAAA
GGAGAACATTGATCGTGAGTATTCTGAGACTGACATCCTTGAGAGCGATGT
CCGTGACAAGAAAAACTTCGCCGTGTTGAAAAAGATCTATCTGAATGAAAA
CGTGAACTCGGAGGAATTGGAAGTTTTTCGTAACGACATTAAGAAGAAACT
GAACAAAATCAACAGCCTGAAGTACTCATTTGAAAAGAATAAGGCGAATTAT
CAAAAGATTAATGAGAATAACATCGAGAAGGTTGAAGGTAAGTCAAAGCGT
AACATTATTTACGATTATTATCGTGAGTCAGCGAAACGTGACGCTTATGTAA
GCAATGTGAAAGAAGCCTTTGATAAGCTTTACAAGGAAGAGGACATTGCAA
AACTTGTTCTTGAAATTGAGAACCTTACGAAGTTAGAGAAATACAAGATTCG
CGAGTTCTACCACGAAATTATTGGACGTAAGAATGACAAGGAAAACTTTGC

AAAAATCATCTACGAAGAAATCCAGAATGTTAATAACATGAAAGAGTTGATC
GAGAAGGTACCGGACATGAGTGAGTTGAAAAAGAGCCAAGTATTTTACAAG
TATTACTTAGACAAAGAAGAGTTGAACGACAAGAACATCAAATACGCGTTTT
GTCATTTCGTGGAAATCGAAATGAGTCAGTTGCTGAAGAACTACGTATATAA
GCGCTTAAGTAATATCTCGAATGACAAAATTAAGCGTATCTTTGAATACCAG
AACTTGAAAAAATTGATCGAAAATAAGCTGTTAAACAAACTTGACACGTACG
TCCGTAATTGTGGAAAGTATAATTATTATTTGCAAGACGGCGAAATTGCCAC
TTCAGATTTCATCGCCCGCAACCGTCAGAATGAAGCGTTTCTTCGCAACAT
CATTGGGGTGTCATCTGTGGCCTACTTTTCTCTTCGCAACATTCTTGAAACG
GAGAACGAGAATGATATTACTGGGCGTATGCGCGGCAAAACAGTTAAGAA
CAATAAAGGTGAAGAGAAGTACGTGTCCGGAGAAGTTGATAAGATCTATAA
TGAAAATAAGAAGAACGAGGTTAAGGAGAACTTAAAAATGTTCTATTCGTAC
GATTTCAATATGGACAACAAGAATGAAATCGAAGATTTCTTCGCCAACATCG
ACGAGGCGATTTCTTCCATCCGTCACGGTATTGTCCACTTCAACTTGGAAT
TAGAAGGTAAGGATATCTTTGCGTTCAAGAACATTGCGCCATCCGAAATCT
CAAAGAAGATGTTTCAGAATGAGATTAACGAGAAAAAACTGAAATTGAAGAT
CTTTCGTCAACTGAACTCTGCCAACGTGTTCCGCTATCTCGAAAAGTATAAA
ATTCTGAATTACCTTAAACGTACACGCTTCGAGTTTGTCAATAAAAATATCC
CATTCGTCCCGTCTTTCACCAAATTATATTCGCGCATTGATGACCTGAAGAA
TAGTCTTGGGATTTACTGGAAAACTCCGAAAACAAACGACGACAATAAGAC
TAAGGAGATTATTGATGCCCAAATCTATTTGCTTAAAAACATCTATTACGGG
GAGTTCCTGAATTATTTCATGTCGAACAATGGTAATTTCTTTGAGATTTCTAA
AGAAATCATCGAATTGAACAAGAACGATAAACGCAACTTAAAGACTGGGTT
TTACAAGCTGCAAAAGTTTGAAGACATCCAGGAGAAGATTCCAAAGGAATA
CTTGGCGAATATCCAGTCCCTGTACATGATTAATGCCGGTAATCAGGACGA
AGAAGAAAAGGACACTTATATTGATTTCATTCAAAAGATCTTCTTAAAGGGA
TTTATGACGTATCTTGCTAATAACGGTCGTTTAAGTCTGATTTACATCGGCT
CGGATGAAGAAACAAATACGTCATTAGCAGAAAAGAAGCAAGAGTTTGACA
AGTTCTTGAAGAAGTACGAGCAGAACAATAATATCAAGATCCCCTATGAGA
TCAATGAATTCCTGCGTGAGATCAAACTGGGAAACATCCTGAAGTATACTG
AGCGTTTAAACATGTTCTACCTTATCTTAAAGCTTTTGAATCACAAGGAGCT
GACAAATCTGAAGGGTAGTCTTGAAAAATATCAGTCTGCCAATAAGGAAGA
AGCGTTCTCTGACCAATTGGAGTTAATTAACCTGCTTAACCTTGACAACAAC
CGCGTGACGGAAGACTTCGAATTAGAGGCCGACGAGATTGGAAAATTTCTT
GATTTCAATGGCAACAAAGTTAAGGATAACAAGGAACTGAAAAAGTTCGATA
CAAACAAGATCTACTTTGACGGCGAGAACATTATCAAACACCGTGCCTTCT
ACAATATTAAGAAATATGGCATGTTAAACTTACTGGAGAAAATTGCCGACAA
GGCTGGATACAAGATCTCGATCGAAGAGCTGAAGAAATACTCCAATAAAAA
GAATGAGATCGAGAAGAACCATAAGATGCAGGAAAATCTGCACCGCAAATA
CGCTCGTCCCCGTAAAGACGAGAAGTTTACAGATGAGGACTATGAAAGTTA
CAAGCAAGCTATTGAGAATATTGAGGAGTACACCCACCTTAAGAACAAGGT
AGAATTCAATGAGCTGAATTTACTGCAGGGCCTGTTGCTGCGCATTTTACAT
CGTTTAGTCGGATATACCTCAATTTGGGAACGCGATCTGCGCTTCCGCCTT
AAAGGTGAGTTCCCAGAAAACCAATACATCGAAGAGATCTTCAACTTTGAA
AATAAGAAGAACGTGAAGTACAAAGGGGGTCAGATTGTAGAGAAATACATT
AAATTCTACAAGGAATTACATCAAAATGATGAAGTTAAGATCAACAAGTACA
GTTCCGCGAATATCAAGGTGTTGAAGCAAGAAAAGAAGGACCTTTATATTC
GaAATTACATCGCCCACTTCAATTATATTCCTCACGCCGAGATCTCACTGCT

GGAAGTCCTTGAAAATTTGCGTAAATTGCTGTCCTACGATCGCAAACTGAA
AAATGCCGTAATGAAATCAGTAGTTGATATCCTTAAGGAGTATGGTTTTGTA
GCCACATTCAAAATCGGGGCGGACAAGAAGATCGGTATTCAGACACTGGA
GAGCGAAAAAATCGTGCATCTTAAGAATCTTAAGAAGAAGTTAATGACT
GACCGCAATTCCGAGGAACTTTGCAAATTGGTGAAGATTATGTTTGAATACA
AAATGGAAGAGAAAAAGTCTGAAAACtaataacATTGGAAGTGGATAACGGAT
CCGCGATCGCGGCGCGCCACCTGGTGGCCGGCCGGTACCACGCGTGCG
CGCTGATCCGGCTGCTAACAAAGCCCGAAAGGAAGCTGAGTTGGCTGCTG
CCACCGCTGAGCAATAACTAGCATAACCCCTTGGGGCCTCTAAACGGGTC
TTGAGGGGTTTTTTGCTGAAAGGAGGAACTATATCCGGATATCCACAGGAC
GGGTGTGGTCGCCATGATCGCGTAGTCGATAGTGGCTCCAAGTAGCGAAG
CGAGCAGGACTGGGCGGCGGCCAAAGCGGTCGGACAGTGCTCCGAGAA
CGGGTGCGCATAGAAATTGCATCAACGCATATAGCGCTAGCAGCACGCCA
TAGTGACTGGCGATGCTGTCGGAATGGACGATATCCCGCAAGAGGCCCG
GCAGTACCGGCATAACCAAGCCTATGCCTACAGCATCCAGGGTGACGGTG
CCGAGGATGACGATGAGCGCATTGTTAGATTTCATACACGGTGCCTGACT
GCGTTAGCAATTTAACTGTGATAAACTACCGCATTAAAGCTTATCGATGATA
AGCTGTCAAACATGAGAATTCTTGAAGACGAAAGGGCCTCGTGATACGCCT
ATTTTTATAGGTTAATGTCATGATAATAATGGTTTCTTAGACGTCAGGTGGCA
CTTTTCGGGGAAATGTGCGCGGAACCCCTATTTGTTTATTTTTCTAAATACA
TTCAAATATGTATCCGCTCATGAGACAATAACCCTGATAAATGCTTCAATAA
CATTGAAAAAGGAAGAGTATGAGTATTCAACATTTCCGTGTCGCCCTTATTC
CCTTTTTTGCGGCATTTTGCCTTCCTGTTTTTGCTCACCCAGAAACGCTGGT
GAAAGTAAAAGATGCTGAAGATCAGTTGGGTGCACGAGTGGGTTACATCG
AACTGGATCTCAACAGCGGTAAGATCCTTGAGAGTTTTCGCCCCGAAGAAC
GTTTTCCAATGATGAGCACTTTTAAAGTTCTGCTATGTGGCGCGGTATTATC
CCGTGTTGACGCCGGGCAAGAGCAACTCGGTCGCCGCATACACTATTCTC
AGAATGACTTGGTTGAGTACTCACCAGTCACAGAAAAGCATCTTACGGATG
GCATGACAGTAAGAGAATTATGCAGTGCTGCCATAACCATGAGTGATAACA
CTGCGGCCAACTTACTTCTGACAACGATCGGAGGACCGAAGGAGCTAACC
GCTTTTTTGCACAACATGGGGGATCATGTAACTCGCCTTGATCGTTGGGAA
CCGGAGCTGAATGAAGCCATACCAAACGACGAGCGTGACACCACGATGC
CTGCAGCAATGGCAACAACGTTGCGCAAACTATTAACTGGCGAACTACTTA
CTCTAGCTTCCCGGCAACAATTAATAGACTGGATGGAGGCGGATAAAGTTG
CAGGACCACTTCTGCGCTCGGCCCTTCCGGCTGGCTGGTTTATTGCTGAT
AAATCTGGAGCCGGTGAGCGTGGGTCTCGCGGTATCATTGCAGCACTGGG
GCCAGATGGTAAGCCCTCCCGTATCGTAGTTATCTACACGACGGGGAGTC
AGGCAACTATGGATGAACGAAATAGACAGATCGCTGAGATAGGTGCCTCA
CTGATTAAGCATTGGTAACTGTCAGACCAAGTTTACTCATATATACTTTAGAT
TGATTTAAAACTTCATTTTTAATTTAAAAGGATCTAGGTGAAGATCCTTTTTGA
TAATCTCATGACCAAAATCCCTTAACGTGAGTTTTCGTTCCACTGAGCGTCA
GACCCCGTAGAAAAGATCAAAGGATCTTCTTGAGATCCTTTTTTTCTGCGC
GTAATCTGCTGCTTGCAAACAAAAAAACCACCGCTACCAGCGGTGGTTTGT
TTGCCGGATCAAGAGCTACCAACTCTTTTTCCGAAGGTAACTGGCTTCAGC
AGAGCGCAGATACCAAATACTGTCCTTCTAGTGTAGCCGTAGTTAGGCCAC
CACTTCAAGAACTCTGTAGCACCGCCTACATACCTCGCTCTGCTAATCCTG
TTACCAGTGGCTGCTGCCAGTGGCGATAAGTCGTGTCTTACCGGGTTGGA
CTCAAGACGATAGTTACCGGATAAGGCGCAGCGGTCGGGCTGAACGGGG

GGTTCGTGCACACAGCCCAGCTTGGAGCGAACGACCTACACCGAACTGA
GATACCTACAGCGTGAGCTATGAGAAAGCGCCACGCTTCCCGAAGGGAGA
AAGGCGGACAGGTATCCGGTAAGCGGCAGGGTCGGAACAGGAGAGCGC
ACGAGGGAGCTTCCAGGGGGAAACGCCTGGTATCTTTATAGTCCTGTCGG
GTTTCGCCACCTCTGACTTGAGCGTCGATTTTTGTGATGCTCGTCAGGGGG
GCGGAGCCTATGGAAAAACGCCAGCAACGCGGCCTTTTTACGGTTCCTGG
CCTTTTGCTGGCCTTTTGCTCACATGTTCTTTCCTGCGTTATCCCCTGATTC
TGTGGATAACCGTATTACCGCCTTTGAGTGAGCTGATACCGCTCGCCGCA
GCCGAACGACCGAGCGCAGCGAGTCAGTGAGCGAGGAAGCGGAAGAGC
GCCTGATGCGGTATTTTCTCCTTACGCATCTGTGCGGTATTTCACACCGCA
TATATGGTGCACTCTCAGTACAATCTGCTCTGATGCCGCATAGTTAAGCCA
GTATACACTCCGCTATCGCTACGTGACTGGGTCATGGCTGCGCCCCGACA
CCCGCCAACACCCGCTGACGCGCCCTGACGGGCTTGTCTGCTCCCGGCA
TCCGCTTACAGACAAGCTGTGACCGTCTCCGGGAGCTGCATGTGTCAGAG
GTTTTCACCGTCATCACCGAAACGCGCGAGGCAGCTGCGGTAAAGCTCAT
CAGCGTGGTCGTGAAGCGATTCACAGATGTCTGCCTGTTCATCCGCGTCC
AGCTCGTTGAGTTTCTCCAGAAGCGTTAATGTCTGGCTTCTGATAAAGCGG
GCCATGTTAAGGGCGGTTTTTTCCTGTTTGGTCACTGATGCCTCCGTGTAA
GGGGGATTTCTGTTCATGGGGGTAATGATACCGATGAAACGAGAGAGGAT
GCTCACGATACGGGTTACTGATGATGAACATGCCCGGTTACTGGAACGTT
GTGAGGGTAAACAACTGGCGGTATGGATGCGGCGGGACCAGAGAAAAAT
CACTCAGGGTCAATGCCAGCGCTTCGTTAATACAGATGTAGGTGTTCCACA
GGGTAGCCAGCAGCATCCTGCGATGCAGATCCGGAACATAATGGTGCAG
GGCGCTGACTTCCGCGTTTCCAGACTTTACGAAACACGGAAACCGAAGAC
CATTCATGTTGTTGCTCAGGTCGCAGACGTTTTGCAGCAGCAGTCGCTTCA
CGTTCGCTCGCGTATCGGTGATTCATTCTGCTAACCAGTAAGGCAACCCC
GCCAGCCTAGCCGGGTCCTCAACGACAGGAGCACGATCATGCGCACCCG
TGGCCAGGACCCAACGCTGCCCGAGATGCGCCGCGTGCGGCTGCTGGA
GATGGCGGACGCGATGGATATGTTCTGCCAAGGGTTGGTTTGCGCATTCA
CAGTTCTCCGCAAGAATTGATTGGCTCCAATTCTTGGAGTGGTGAATCCGT
TAGCGAGGTGCCGCCGGCTTCCATTCAGGTCGAGGTGGCCCGGCTCCAT
GCACCGCGACGCAACGCGGGGAGGCAGACAAGGTATAGGGCGGCGCCT
ACAATCCATGCCAACCCGTTCCATGTGCTCGCCGAGGCGGCATAAATCGC
CGTGACGATCAGCGGTCCAGTGATCGAAGTTAGGCTGGTAAGAGCCGCG
AGCGATCCTTGAAGCTGTCCCTGATGGTCGTCATCTACCTGCCTGGACAG
CATGGCCTGCAACGCGGGCATCCCGATGCCGCCGGAAGCGAGAAGAATC
ATAATGGGGAAGGCCATCCAGCCTCGCGTCGCGAACGCCAGCAAGACGT
AGCCCAGCGCGTCGGCCGCCATGCCGGCGATAATGGCCTGCTTCTCGCC
GAAACGTTTGGTGGCGGGACCAGTGACGAAGGCTTGAGCGAGGGCGTGC
AAGATTCCGAATACCGCAAGCGACAGGCCGATCATCGTCGCGCTCCAGC
GAAAGCGGTCCTCGCCGAAAATGACCCAGAGCGCTGCCGGCACCTGTCC
TACGAGTTGCATGATAAAGAAGACAGTCATAAGTGCGGCGACGATAGTCAT
GCCCCGCGCCCACCGGAAGGAGCTGACTGGGTTGAAGGCTCTCAAGGGC
ATCGGTCGACGCTCTCCCTTATGCGACTCCTGCATTAGGAAGCAGCCCAG
TAGTAGGTTGAGGCCGTTGAGCACCGCCGCCGCAAGGAATGGTGCATGC
AAGGAGATGGCGCCCAACAGTCCCCCGGCCACGGGGCCTGCCACCATAC
CCACGCCGAAACAAGCGCTCATGAGCCCGAAGTGGCGAGCCCGATCTTC
CCCATCGGTGATGTCGGCGATATAGGCGCCAGCAACCGCACCTGTGGCG

CCGGTGATGCCGGCCACGATGCGTCCGGCGTAGAGGATCGAGATCTCGA<br>TCCCGCGAAT

iSpinach DNA template<br>https://benchling.com/s/seq-4ZEaWlbno3qfH2Il0MgK<br>T7 promoter, iSpinach coding sequence
tgccacctgacgtctaagaaCCTTAATACGACTCACTATAGGGccataccaaacgacgag<br>cgtgacGGGAGTACGGTGAGGGTCGGGTCCAGTAGCTTCGGCTACTGTTGA<br>GTAGAGTGTGGGCTCCGTACTCCCgtcacgctcgtcgtttggtagctcactcaaaggcggt<br>aat

crRNA DNA template, specific for Lbu Cas13a and *E. coli* RNA target<br>https://benchling.com/s/seq-9tO076KKpnMBWk4Tptcf<br>T7 promoter, Lbu scaffold, target complementary sequence
gtgtgagctcctaatacgactcactataggggaccaccccaaaaatgaaggggactaaaacaACTTTA<br>CTCCCTTCCTCCCCGCTGAAAGat

Cognate targetRNA DNA template, sub-sequence of *E. coli* 16S rRNA<br>https://benchling.com/s/seq-7taI0lXQ3f8a2ircGWWc<br>T7 promoter, crRNA complementary sequence
tgccacctgacgtctaagaaagtgtgagctcctaatacgactcactataggCAAGCCTGATGCAG<br>CCATGCCGCGTGTATGAAGAAGGCCTTCGGGTTGTAAAGTACTTTCAGCG<br>GGGAGGAAGGGAGTAAAGTTAATACCTTTGCTCATTGACGTTACCCGCAGA<br>AGAAGCACCGGCTAAgctcactcaaaggcggtaat

Non-cognate targetRNA DNA template, sub-sequence of *B. subtilis* 16S rRNA<br>https://benchling.com/s/seq-1qIllvBpAFT35ptsmbSm<br>T7 promoter
tgccacctgacgtctaagaaagtgtgagctcctaatacgactcactataggAAGTCTGACGGAG<br>CAACGCCGCGTGAGTGATGAAGGTTTTCGGATCGTAAAGCTCTGTTGTTAG<br>GGAAGAACAAGTACCGTTCGAATAGGGCGGTACCTTGACGGTACCTAACC<br>AAAAAGCCACGGCTAAgctcactcaaaggcggtaat
